# Delayed Iatrogenic Direct Carotid Cavernous Fistula Following Flow Diversion for Aneurysm With Spontaneous Healing: A Case Report

**DOI:** 10.7759/cureus.58944

**Published:** 2024-04-24

**Authors:** Evelyn B Voura, John R Stulb, Jorge L Eller, David J Padalino, Raghu Ramaswamy

**Affiliations:** 1 Department of Neurosurgery, Crouse Neuroscience Institute, Crouse Health at Crouse Hospital, Crouse Medical Practice, PLLC, Syracuse, USA; 2 Department of Neuroscience and Physiology, State University of New York Upstate Medical University, Syracuse, USA

**Keywords:** spontaneous healing, iatrogenic, pipeline stent, flow diversion, carotid cavernous fistula

## Abstract

An abnormal connection between the carotid artery and cavernous sinus is referred to as a carotid cavernous fistula (CCF). A direct CCF results when the connection occurs between the intracranial internal carotid artery (ICA) and the cavernous sinus. These events are typically the result of a head injury, but can also be iatrogenic, resulting from various intracranial procedures. Direct CCF occurrences rarely heal spontaneously due to the high flow rate across the fistula. In this report, we present an uncommon case involving a delayed iatrogenic direct CCF, which developed following the placement of a pipeline flow-diverting stent that was used to treat a cerebral aneurysm. Interestingly, this unusual iatrogenic direct CCF subsequently spontaneously resolved within a few months. To our knowledge, this is the only case of a delayed CCF occurring with the use of a flow-diverting sent, which then resolved on its own. This report recounts our experience with the case.

## Introduction

A carotid cavernous fistula (CCF) is an abnormal connection between the carotid artery and the cavernous sinus [1-3]. It is diagnosed with cerebral imaging (angiography is preferred) and is classified based on the flow rate (high flow and low flow), etiology (spontaneous, traumatic, and iatrogenic), and by the anatomic feeders (direct and indirect) [2]. High-flow fistulas are more common and aggressive than those that are low-flow [1,2]. Flow rates often depend on the etiology. Traumatic and iatrogenic fistulas tend to have high flow due to the associated often direct tear in the cavernous artery wall causing the fistula [1-4]. Iatrogenic occurrences have been reported after a variety of procedures including transsphenoidal pituitary surgery, balloon angioplasty, tumor embolization, carotid stenting, aneurysm coiling, and mechanical thrombectomy [5-12].

While rare, spontaneous healing of CCF events has been reported [4,13,14]. While the mechanism of spontaneous healing is unknown, it has been suggested that spontaneous closure is aided by thrombosis within CCF events having certain etiologies that are typically associated with a low-flow classification [1,4]. Conservative management of low-flow CCFs may lead to spontaneous occlusion, but this approach is generally less effective for high-flow CCF cases [2]. Due to the high-flow rate across the fistula, direct CCFs generally require treatment because they rarely heal spontaneously [4]. Furthermore, the high flow rate can result in various visual detriments when direct CCFs are left untreated due to possible raised intraocular pressure, compromised ocular motor nerve function, and orbital congestion which can detrimentally affect vision in the long run [3,11]. Consequently, if managing a CCF conservatively, careful monitoring of vision is suggested [1,4]. Endovascular treatment using different modalities offers a greater than 80% success rate, with complete recovery in most instances [2,3,11,15]. The successful endovascular treatment of direct CCFs depends on the specific flow conditions and the anatomy of the fistula. Flow diversion is increasingly being used to reroute the blood away from the cavernous sinus, with positive results being reported [2,3,16]. Regardless of the technique, the treatment goal is to preserve blood flow through the internal carotid artery (ICA) while completely occluding the fistula [2]. In this article, we present an unusual case of an iatrogenic CCF with delayed development following the placement of a pipeline flow-diverting stent to treat a cerebral aneurysm, which then unexpectedly spontaneously resolved prior to treatment.

## Case presentation

**Figure 1:**
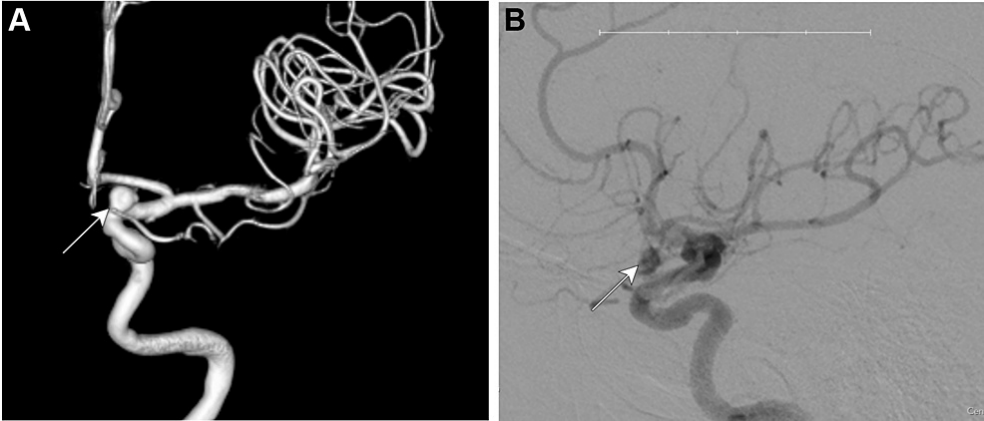
Angiogram prior to treatment of the aneurysm. This imaging was completed in December 2019. The images show the left ophthalmic ICA aneurysm with dysmorphic features having a daughter sac (arrows) following a left ICA injection and cerebral run, (A) from an anterior-posterior view with a 3D reconstruction and (B) with a lateral 2D view of the lesion. The images were taken using a Toshiba Infinix and Omnipaque 300 (Chicago, IL: GE Healthcare) as a contrast agent. ICA: internal carotid artery

In 2016, a 56-year-old female patient with dizziness was referred to our neurosurgery service from another facility. Other than smoking, she had no significant past medical history. Our investigation into the cause of her symptoms revealed a left paraophthalmic internal carotid artery (ICA) aneurysm. She underwent a cerebral angiogram which confirmed a left paraophthalmic ICA aneurysm measuring 3.6 mm. Conservative management with follow-up surveillance imaging was recommended. Her next cerebral vascular imaging in December 2019 indicated that the aneurysm had increased in size (Figures 1A, 1B).

**Figure 2:**
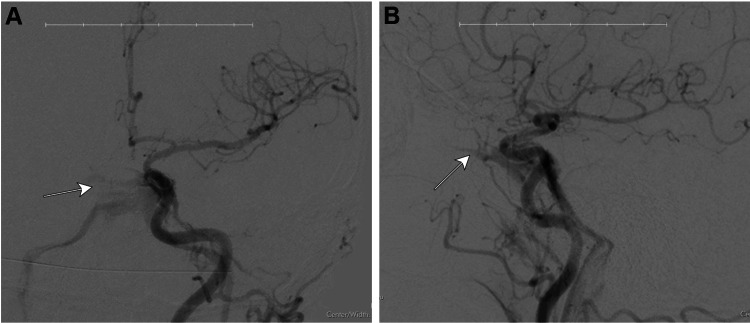
Six-month post-aneurysm treatment follow-up angiogram. The angiography completed in July 2020, which was six months after the treatment of the left ophthalmic ICA aneurysm using a 3x16 mm pipeline flow diverting stent (Minneapolis, MN: Medtronic), indicated that the left ophthalmic ICA aneurysm treated with the stent had resolved. However, a likely iatrogenic, directly left carotid cavernous fistula with venous drainage into both cavernous sinuses and both inferior petrosal sinuses was observed (arrows). The images show the lesion following a left common carotid injection and cerebral run, 2D (A) anterior-posterior and (B) lateral views. The images were taken using a Toshiba Infinix and Omnipaque 300 (Chicago, IL: GE Healthcare) as a contrast agent. ICA: internal carotid artery

Repeat angiography completed several weeks later (in January 2020) confirmed that the aneurysm had expanded - measuring 5x4.9 mm - and that it also had some dysmorphic attributes. Due to the increased size of the aneurysm, the presence of dysmorphic features and continued smoking by the patient, endovascular intervention in the form of flow diverting stent was recommended. Since the ophthalmic artery came off the neck of the aneurysm, primary coiling would have had to be incomplete, or otherwise compromise the artery. Therefore, flow diversion was the safest option for our patient. She underwent pipeline embolization for the ICA aneurysm in January 2020. Immediate post-procedure angiography confirmed the excellent position of the stent with no obvious complications. Her immediate post-procedure stay was one day as per our protocol and was uneventful. She then had a two-week post-procedure follow-up appointment at which time she reported reduced headaches and no new symptoms. During the preparation for her six-month follow-up visit and angiogram, she mentioned hearing a “swishing sound” in her left ear. The scheduled six-month post-procedure cerebral angiography took place in July 2020. The new imaging revealed a direct CCF arising at the proximal end of the stent with venous drainage into both cavernous sinuses and posterior drainage into the inferior petrosal sinuses on both sides (Figures 2A, 2B).

**Figure 3:**
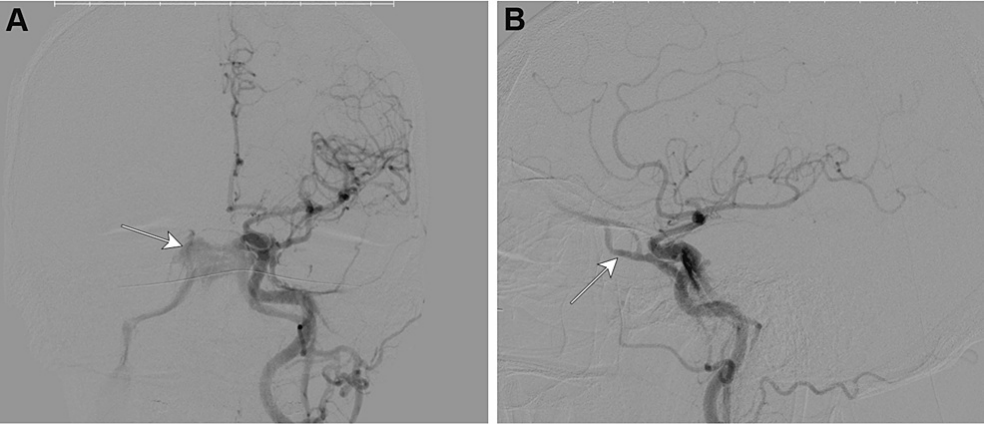
Ten-month post-aneurysm treatment follow-up angiogram. Angiography completed in November 2020, which was 10 months post-treatment of the left ophthalmic ICA aneurysm using a 3x16 mm pipeline flow diverting stent (Medtronic, Minneapolis, MN), revealed a persisting left carotid cavernous fistula with venous drainage into both cavernous sinuses and both inferior petrosal sinuses (arrows). At this time, we observed increased drainage anteriorly into the ophthalmic vein compared to the previous angiogram from July 2020. The images show the lesion following a left common carotid artery injection and cerebral run, 2D (A) anterior-posterior and (B) lateral views. The images were taken using a Toshiba Infinix and Omnipaque 300 (Chicago, IL: GE Healthcare) as a contrast agent. ICA: internal carotid artery

As she was asymptomatic apart from the swishing sounds in her left ear, we decided to manage this development conservatively with a plan for repeat angiography after six months. The follow-up imaging occurred in November 2020 and indicated continued flow through CCF with drainage into both cavernous sinuses and the inferior petrosal sinuses with increased flow into the superior and inferior orbital veins (Figures 3A, 3B).

**Figure 4:**
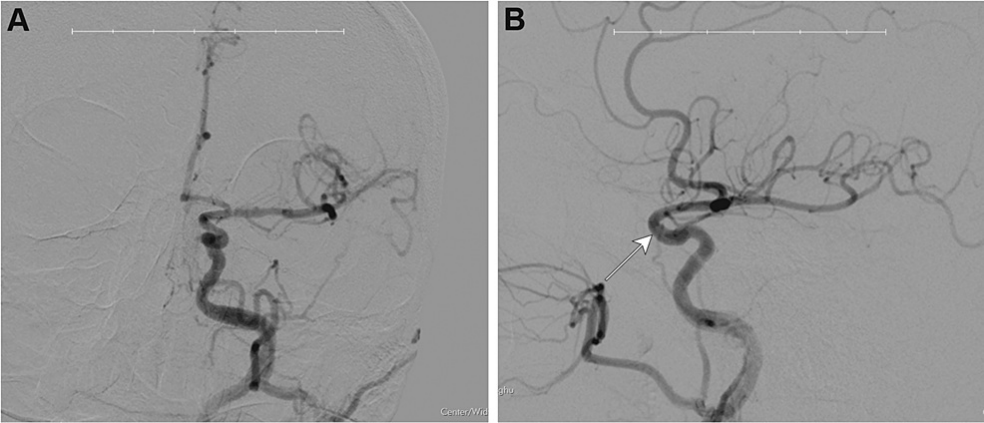
Fourteen-month post-aneurysm treatment follow-up angiogram. This angiography was completed in March of 2021, which was 14 months post-treatment of the left ophthalmic ICA aneurysm using a 3x16 mm pipeline flow diverting stent (Minneapolis, MN: Medtronic). At this time, we observed complete spontaneous resolution of the left-sided carotid cavernous fistula. The arrow indicates where we would have expected to see CCF based on our previous imaging results. There is no residual/recurrence of the aneurysm seen on this angiogram. The images show the area following a left common carotid artery injection and cerebral run, 2D (A) anterior-posterior and (B) lateral views. The images were taken using a Toshiba Infinix and Omnipaque 300 (Chicago, IL: GE Healthcare) as a contrast agent. ICA: internal carotid artery

Since the anterior drainage into the orbital veins had increased, treatment for CCF was recommended. A transarterial approach for another pipeline stent as treatment of CCF, with a transvenous approach for coil/onyx embolization of the cavernous and orbital venous channels, was planned since the use of a pipeline stent alone would likely have been insufficient for this high-flow CCF. The procedure was delayed until March 2021 due to restrictions on elective cases imposed at our institution as a result of the COVID-19 pandemic. Cerebral angiography during the planned treatment then showed complete spontaneous resolution of CCF so the procedure was discontinued (Figures 4A, 4B). Consequently, her prescription for Plavix was stopped and she is now prescribed a small dose of aspirin (81 mg/day).

## Discussion

A variety of intracranial procedures can result in the formation of an iatrogenic direct CCF [5,12,17]. Since these lesions typically do not heal spontaneously, direct CCFs generally require treatment to avoid visual deficits [3,4]. Despite this precedent, we presented a case of a delayed CCF occurrence following the placement of a flow-diverting stent to treat an aneurysm, which subsequently resolved without intervention.

To understand the indicators that might predict the risk of an iatrogenic CCF complication, some groups have studied the circumstances of these cases at their institutions. Yeh et al. studied 138 surgical interventions to treat chronic carotid artery occlusion and observed that 8% of these resulted in a CCF [18]. The instances were self-limiting, and none resulted in stroke or death. As a result, they recommended that CCF cases be managed conservatively. In comparison, Ono et al. studied 1,071 endovascular procedures and observed that only 0.8% resulted in a CCF [19]. Both groups noted a higher proportion of female patients in CCF-positive patient cohorts, consistent with the previous observations reported by Barrow et al. [1].

While infrequent, there are reports of spontaneous high-flow CCF resolution. One report documented a case of a healed CCF that formed as a result of a motor vehicle accident [14]. Iampreechakul et al., reported on nine other cases of direct CCFs that resolved without treatment - two of these cases were spontaneous, but the other seven resulted from some form of trauma [4]. This group also described another 37 examples from the literature, many of which were spontaneous in nature or resulted from an identified trauma and healed with time without intervention. Interestingly, a number of these incidents such as one reported by Voigt et al., reported that their CCF cases closed following carotid angiography [13]. Iampreechakul et al. also identified two other cases reported by Kwon and Jin that were more akin to this case in that they were iatrogenic in nature [8]. One of these cases occurred after the placement of a stent to prevent the reoccurrence of a subarachnoid hemorrhage, and the other after stenting and coiling for the treatment of an aneurysm. Both lesions closed without treatment a week and one month later, for the hemorrhage and the aneurysm patients, respectively. We are aware of only one other related iatrogenic CCF report authored by Park et al., wherein stent-assisted coiling for the treatment of an aneurysm resulted in the formation of a CCF, which then also resolved itself [20]. While our case was similar to these other reports in that it spontaneously healed, it differed in that we used a pipeline flow diverting stent and also observed the delayed formation of CCF (first noted at the six-month follow-up appointment). We are uncertain of the reason for the delayed formation of CCF in our patient, and, as with the other reports, the circumstances leading to the spontaneous resolution of CCF are also unknown. As mentioned above, it is hypothesized that CFF resolution may be facilitated by thrombus formation [1,4]. In our case, Plavix was stopped six months after the initial treatment but was restarted in anticipation of placing the flow-diverting stent to manage CCF. We believe the observations made with our case to be rare in that we have not found another report of a delayed onset CCF occurring with the use of a flow-diverting sent, which went on to spontaneously resolve.

## Conclusions

With the steady increase in the application of endovascular procedures, iatrogenic CCF occurrences will likely become more common. Therefore, it is important to recognize cases of iatrogenic CCF that have delayed development and also acknowledge that these, as we and others observed, may also spontaneously heal. Flow rate has been theorized to be the determining factor governing the likelihood of spontaneous closure and successful conservative management of CCFs. This case may suggest that direct CCFs associated with flow-diverting stents may have a greater chance of spontaneous closure despite their higher rate of flow. This may be due to the flow dynamics that occur within and adjacent to the stent.
